# Stereo Gaussian Splatting with Adaptive Scene Depth Estimation for Semantic Mapping

**DOI:** 10.3390/jimaging12030105

**Published:** 2026-02-28

**Authors:** Chenhui Fu, Jiangang Lu

**Affiliations:** State Key Laboratory of Industrial Control Technology, College of Control Science and Engineering, Zhejiang University, Hangzhou 310027, China; chfu@zju.edu.cn

**Keywords:** semantic SLAM, 3D Gaussian splatting, explicit representation

## Abstract

Simultaneous Localization and Mapping (SLAM) is a fundamental capability in robotics and augmented reality. However, achieving accurate geometric reconstruction and consistent semantic understanding in complex environments remains challenging. Although recent neural implicit representations have improved reconstruction quality, they often suffer from high computational cost and the forgetting phenomenon during online mapping. In this paper, we propose StereoGS-SLAM, a stereo semantic SLAM framework based on 3D Gaussian Splatting (3DGS) for explicit scene representation. Unlike existing approaches, StereoGS-SLAM operates on passive RGB stereo inputs without requiring active depth sensors. An adaptive depth estimation strategy is introduced to dynamically refine Gaussian scales based on real-time stereo depth estimates, ensuring robust and scale-consistent reconstruction. In addition, we propose a hybrid keyframe selection strategy that integrates motion-aware selection with lightweight random sampling to improve keyframe diversity and maintain stable, real-time optimization. Experimental evaluations demonstrate that StereoGS-SLAM achieves consistent and competitive localization, rendering, and semantic reconstruction performance compared with recent 3DGS-based SLAM systems.

## 1. Introduction

With the rapid development of robotics and augmented reality, SLAM systems have received increasing attention in recent years. Traditional SLAM methods [1,2,3] primarily rely on sparse feature points or geometric primitives to represent the environment. Although they offer high computational efficiency, these methods often struggle to provide rich semantic understanding and accurate geometric reconstruction in complex and dynamic environments. To overcome these limitations, researchers have explored the integration of semantic information into SLAM frameworks to enhance environmental perception and system robustness.

3DGS has recently emerged as an explicit and differentiable scene representation technique in the SLAM domain [4]. Compared with traditional implicit methods, 3DGS enables faster rendering and higher reconstruction accuracy by allowing gradients to flow directly to the parameters of each Gaussian [5], establishing an almost linear relationship between photometric loss and Gaussian parameters during optimization. However, existing 3DGS-based SLAM systems predominantly depend on RGB-D sensors for depth acquisition, which limits their deployment in scenarios where active depth sensors are unavailable. Additionally, while recent studies have attempted to incorporate semantic information into the 3DGS framework, challenges remain in maintaining semantic consistency and spatial alignment, particularly in scenes with large depth variations.

To address these issues, this paper proposes StereoGS-SLAM, a stereo semantic 3DGS SLAM system. Our method utilizes a deep stereo matching network to predict dense depth maps from passive stereo RGB inputs, eliminating the need for active depth sensing. An adaptive scene depth estimation strategy dynamically adjusts the Gaussian scales based on real-time stereo depth estimates, enhancing reconstruction quality and robustness across diverse depth ranges. Furthermore, semantic information is integrated into the Gaussian optimization process to improve both semantic consistency and spatial alignment accuracy. The main contributions of this work are summarized as follows:

- We present StereoGS-SLAM, a system-level integration of stereo depth, 3DGS mapping, and semantic optimization that operates exclusively on passive stereo pairs without requiring active depth sensing;
- We design an adaptive scene depth estimation strategy that refines Gaussian scales and improves robustness under large depth variations, emphasizing scale stability within the existing rendering pipeline;
- We develop a hybrid keyframe selection mechanism that combines motion-triggered and fixed-interval policies with stochastic keyframe sampling to improve keyframe diversity and reduce optimization bias.

## 2. Related Work

### 2.1. Traditional Dense Semantic SLAM

Traditional dense semantic SLAM systems typically adopt explicit 3D structures (e.g., voxels, surfels, point clouds, or signed distance fields) to fuse geometry and semantics over time. SLAM++ [3] represents scenes with object-level models, while Co-Fusion [6] and SemanticFusion [7] maintain dense maps with object segmentation and CNN-based semantics. VSO [8] exploits semantics to improve robustness, and QuadricSLAM [9] introduces compact quadric landmarks. Despite their interpretability and mature fusion pipelines, these methods often rely on memory-intensive map representations and careful engineering to maintain real-time performance at high resolution. Moreover, many dense pipelines benefit strongly from direct depth measurements (RGB-D or depth sensing) to stabilize reconstruction; when depth is unavailable or unreliable, maintaining globally consistent dense geometry becomes substantially harder. As a result, extending such systems to passive stereo settings while preserving both geometric fidelity and semantic consistency remains non-trivial.

### 2.2. Neural Implicit and 3DGS SLAM

Neural implicit methods, particularly NeRF, model scenes continuously with MLPs but typically require expensive ray-based rendering and optimization, which limits real-time dense mapping and exacerbates online failure modes such as catastrophic forgetting [10]. Systems such as iMAP [11], NICE-SLAM [12], ESLAM [13], Vox-Fusion [14], and GO-SLAM [15] improve scalability, yet their performance is still often bounded by the computational cost of implicit rendering and the sensitivity of incremental optimization.

3D Gaussian Splatting (3DGS) provides an explicit, differentiable representation with efficient splatting-based rendering [4], making it attractive for SLAM. Compared with NeRF-style implicit models, 3DGS avoids costly volumetric rendering and enables faster optimization with explicit primitives; compared with voxel-based methods, it offers a more compact continuous representation without dense voxel grids, while still supporting efficient rendering. MonoGS [16] leverages monocular depth priors, SplaTAM [5] adopts parallel tracking/mapping for efficiency, and GS-SLAM [17] incorporates monocular depth for scale-related initialization. Semantic extensions such as GS3LAM [18], NEDS-SLAM [19], SDD-SLAM [20], and GSORB-SLAM [21] further integrate semantic cues into Gaussian maps. However, existing Gaussian-based SLAM pipelines still depend on RGB-D inputs or learned monocular depth, which restricts deployment in scenarios without active depth sensors or with poor depth generalization. In addition, 3DGS optimization is sensitive to scale initialization: large depth-range variations across frames can lead to over- or undersized Gaussians, degrading convergence stability and reconstruction fidelity, yet scale-consistent mapping under such variations is less explored in prior work.

Overall, these limitations motivate a stereo-only 3DGS semantic SLAM framework that avoids reliance on active depth sensing and enforces scale consistency via adaptive depth-driven Gaussian scaling under large depth-range variations. This work aims to advance recent 3DGS-based SLAM pipelines by addressing specific practical limitations in stereo-only deployment and scale stability.

## 3. Method

**Figure 1:**
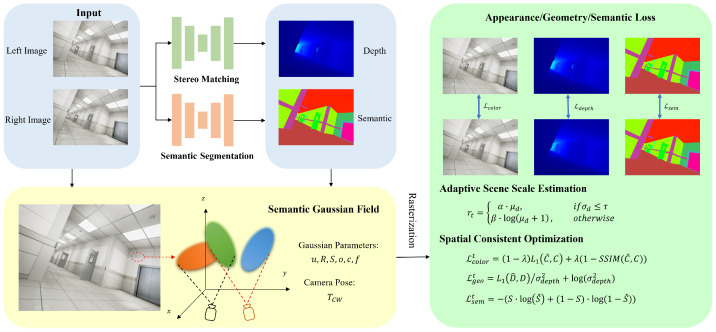
Overview of the StereoGS-SLAM framework. Stereo image pairs are processed by pre-trained stereo and semantic networks to initialize 3D Gaussians with dense depth and semantic cues. Adaptive scene depth estimation dynamically refines Gaussian scales. The system jointly renders color, depth, semantic, and silhouette maps while maintaining geometric and semantic consistency without active depth sensors.

The overview of our proposed StereoGS-SLAM system is illustrated in Figure 1, and the algorithmic formulation is summarized in Algorithm 1. Given a set of stereo images, we first employ a pre-trained stereo network [22] to estimate dense disparity images. The network is pre-trained on the SceneFlow dataset and further fine-tuned on target datasets. During SLAM operation, the network parameters are kept frozen to ensure real-time performance while providing robust depth estimates. Based on the estimated disparity images and the camera intrinsic parameters, dense depth maps are subsequently computed. These maps offer rich geometric guidance for the 3DGS optimization process, benefiting from their high spatial density and more complete scene coverage.
**Algorithm 1** StereoGS-SLAM Overview**Require:** Stereo image stream $\left\{I_{t}^{L},I_{t}^{R}\right\}$, camera intrinsics, pre-trained stereo and semantic networks**Ensure:** Camera poses $\left\{T_{t}\right\}$ and Gaussian map G  1:Initialize G←∅, set $T_{0}$, select initial keyframe set K  2:**for** each time step *t* **do**  3:    Estimate disparity and depth; compute depth statistics $\left(\mu_{d},\sigma_{d}\right)$  4:    Initialize Gaussians $G_{i}=\left(\mu_{i},\Sigma_{i},o_{i},c_{i},f_{i}\right)$ from depth and colors  5:    Render $\hat{C},\hat{D},\hat{F},\hat{O}$ from G and $T_{t}$  6:    **Tracking:** optimize $T_{t}$ by minimizing $L_{tracking}$  7:    Update keyframe set K using the hybrid selection strategy  8:    **Mapping:** sample $K_{t}\subset K$ and optimize G via $L_{color}^{t},L_{geo}^{t},L_{sem}^{t}$  9:    Update semantic decoder $F_{cnn}$ and semantic features $f_{i}$10:    Apply adaptive scene depth estimation to refine Gaussian scales11:**end for**

### 3.1. 3D Gaussian Scene Representation

We generate the dense Gaussian scene representation following the method in [4],

(1) $$G=\{G_{i}:\left(\mu_{i},\Sigma_{i},o_{i},c_{i},f_{i}\right)|i=1,\ldots ,N\}$$

where *N* is the number of Gaussians. Each Gaussian $G_{i}$ is defined by its 3D position $\mu_{i}\in R^{3}$ in the world coordinate system, 3D covariance matrix $\Sigma_{i}\in R^{3\times 3}$, opacity $o_{i}\in \left[0,1\right]$, RGB color $c_{i}\in {\left[0,1\right]}^{3}$, and semantic feature $f_{i}\in R^{N_{sem}}$ ($N_{sem}$ denotes the number of objects in the scene).

With the 3D Gaussian scene representation parameters, we render multiple modalities at each pixel using the differentiable Gaussian splatting technique [4], including color, depth, and semantic features. Given the camera pose $T_{cw}\in SE\left(3\right)$, the *i*-th 3D Gaussian is projected onto the 2D image plane for rendering, with

(2) $$\Sigma_{i}^{\prime }=J_{i}R_{cw}\Sigma_{i}R_{cw}^{T}J_{i}^{T}$$

where $J_{i}\in R^{2\times 3}$ is the Jacobian matrix of the projection function of the *i*-th Gaussian, and $R_{cw}$ is the rotation part of the camera pose $T_{cw}$. After projection, the color of a single pixel is rendered by sorting the Gaussians in depth order and performing front-to-back α-blending,

(3) $$\hat{C}=\sum_{i\in N}c_{i}\alpha_{i}\prod_{j=1}^{i-1}\left(1-\alpha_{j}\right)$$

where $c_{i}$ represents the color of the *i*-th Gaussian, $\alpha_{i}$ is the density computed by the opacity $o_{i}$ and the 2D covariance matrix $\Sigma_{i}^{\prime }$ as,

(4) $$\alpha_{i}=o_{i}\cdot exp\left(-\frac{1}{2}\sigma_{i}^{T}{\left(\Sigma_{i}^{\prime }\right)}^{-1}\sigma_{i}\right)$$

where $\sigma_{i}\in R^{2}$ is the offset vector from the pixel center to the projected mean of the *i*-th Gaussian. Similarly, the depth at each pixel is rendered as,

(5) $$\hat{D}=\sum_{i\in N}d_{i}\alpha_{i}\prod_{j=1}^{i-1}\left(1-\alpha_{j}\right)$$

where $d_{i}$ denotes the depth of the *i*-th Gaussian centroid in the camera coordinate system.

Compared with 3D semantic information, the 2D semantic label is more accessible prior. Additionally, it is memory-inefficient to directly store a high-dimensional semantic feature vector in each Gaussian. Instead, we follow the same procedure as color and depth rendering. The semantic feature at each pixel is computed as,

(6) $$\hat{F}=\sum_{i\in N}f_{i}\alpha_{i}\prod_{j=1}^{i-1}\left(1-\alpha_{j}\right)$$

where $f_{i}\in R^{N_{sem}}$ represents the semantic feature vector for the *i*-th Gaussian, with $N_{sem}$ denoting the number of semantic classes. The semantic features are initialized from semantic segmentation predictions produced by the segmentation network [23] on RGB images, which provide the semantic signals used during online operation.

To decode the semantic label at each pixel, we utilize a lightweight CNN decoder $F_{cnn}$ to map the aggregated semantic features to semantic predictions,

(7) $$\hat{S}=softmax\left(F_{cnn}\left(\hat{F}\right)\right)$$

where the semantic decoder $F_{cnn}$ is implemented as a single convolutional layer that transforms the input semantic feature dimension to the number of semantic classes. The decoder is trained end-to-end with the Gaussian optimization process. Specifically, the decoder weights are initialized randomly and updated jointly with Gaussian semantic features $f_{i}$ using the Adam optimizer. The semantic decoder and Gaussian semantic features are optimized simultaneously to ensure consistency between the 3D scene representation and semantic understanding. This joint optimization strategy allows the system to refine initial SAM-based semantic predictions based on the reconstructed 3D scene geometry and photometric consistency, leading to more accurate and spatially coherent semantic segmentation results.

We encode semantic information directly in the Gaussian primitives via the learnable features $f_{i}$, which are initialized from 2D semantic predictions and then optimized jointly with geometry. During rendering, the semantic features are splatted and composited using the same opacity-aware accumulation as color and depth, so each pixel receives a weighted mixture of nearby Gaussians along the ray. This effectively spreads semantic cues across neighboring primitives while preserving spatial coherence through the Gaussian support and the visibility weights. The subsequent decoder $F_{cnn}$ maps the aggregated feature to class probabilities, and the semantic loss provides feedback that updates both $f_{i}$ and the decoder, encouraging consistent semantic labels across the map.

We also render a silhouette image to determine the cumulative opacity $\hat{O}$ for each pixel,

(8) $$\hat{O}=\sum_{i\in N}\alpha_{i}\prod_{j=1}^{i-1}\left(1-\alpha_{j}\right)$$

### 3.2. Adaptive Scene Depth Estimation

The variations in depth range across different frames result in significant variance in the 3D Gaussian scales corresponding to these frames, leading to overly large or small Gaussian spheres that degrade reconstruction quality and system robustness. To address this limitation, we propose a novel adaptive scene depth estimation strategy. This strategy dynamically estimates and updates the scene depth, enabling more accurate Gaussian initialization and optimization.

For each incoming frame, we derive robust geometric characteristics from the depth distribution to characterize the scene properties. Based on these depth characteristics, we formulate an adaptive scene radius computation that accounts for both local scene characteristics and global scale variations. The adaptive scaling mechanism employs different strategies for uniform and complex scenes: for relatively uniform depth distributions, we use linear scaling of central depth values, while for scenes with significant depth variations, we apply logarithmic transformation to balance the scale estimation for both near and far objects. The adaptive scene radius computation is defined as,    

(9) $$r_{t}=\left\{\begin{array}{cc} \alpha \cdot \mu_{d}, & \mathrm{if}\;\sigma_{d}\leq \tau \\ \beta \cdot \log(\mu_{d}+1), & \mathrm{otherwise} \end{array}\right.$$

where $\mu_{d}$ is the median depth, $\sigma_{d}$ is the depth standard deviation, τ is a scene depth threshold, and α=1.5,β=2 are scaling parameters.

To ensure temporal consistency and prevent abrupt scale variations that could destabilize the system, we apply a temporal smoothing mechanism based on an exponential moving average with a smoothing factor γ=0.7, which balances responsiveness and stability. The resulting adaptive scene depth estimate is then integrated into the Gaussian mapping pipeline. Specifically, during Gaussian scale initialization, the mean squared distances are modulated using the estimated scene depth. This strategy allows the system to effectively handle scenes with varying depth ranges and remain robust under sparse or noisy depth measurements. As the scene depth range evolves over time, the mean Gaussian scale is designed to adapt accordingly rather than strictly converge, reflecting the intended response to changing scene geometry.

### 3.3. Spatial Consistency Mapping

Initial Gaussians are generated from all pixels, as the rendered silhouette map is initially empty. Each Gaussian is initialized with the following properties: color sampled from the corresponding pixel, center position determined by unprojecting the stereo-estimated depth, opacity fixed at 0.5, semantic features randomly initialized using spherical harmonics representation, and radius initialized based on the adaptive scene depth estimation to maintain appropriate projection size in the image plane.

After tracking a frame, we enforce spatial consistency in the Gaussian representation through an adaptive expansion strategy. New Gaussians are selectively introduced only in spatial regions that are inadequately represented by existing elements, ensuring comprehensive yet efficient scene coverage. Spatial consistency is maintained by utilizing cumulative opacity and depth information to construct an unobservable region mask $M_{unobs}$,

(10) $$M_{unobs}=\Theta \left(\hat{O}<\tau_{unobs}\right)\vee I\left(L_{1}\left(\hat{D},D\right)>50\tilde{L_{1}}\left(\hat{D},D\right)\right)$$

where Θ denotes the indicator function, $\tau_{unobs}$ represents the cumulative opacity threshold for unobservable regions, $L_{1}$ indicates the $l_{1}$-norm, and $\tilde{L_{1}}$ corresponds to the median $l_{1}$-norm error between rendered and stereo-estimated depth.

The mapping objective aims to produce a spatially coherent and detailed 3D representation consistent across all observed frames. This is achieved through joint optimization of all Gaussian parameters by minimizing a comprehensive rendering-based objective function. To reduce optimization bias and enhance global map consistency, we employ a random keyframe sampling strategy during mapping. Instead of optimizing all keyframes in each iteration, we randomly sample a subset of keyframes, which discourages overfitting to recent observations and maintains a more balanced representation of the scene. The color loss $L_{color}^{t}$ combines $l_{1}$-norm with structural similarity (SSIM) between rendered and observed colors,

(11) $$L_{color}^{t}=\left(1-\lambda \right)L_{1}\left(\hat{C},C\right)+\lambda \left(1-\mathrm{SSIM}\left(\hat{C},C\right)\right)$$

where $\hat{C}$ and *C* represent rendered and ground truth colors, respectively, with λ=0.2 as the balancing factor.

The geometric loss $L_{geo}^{t}$ incorporates depth uncertainty to enhance the geometric accuracy of the reconstructed scene,    

(12) $$L_{geo}^{t}=\frac{L_{1}\left(\hat{D},D\right)}{\sigma_{depth}^{2}}+\log\left(\sigma_{depth}^{2}\right)$$

where $\hat{D}$ and *D* denote the rendered and estimated depth, respectively, and $\sigma_{depth}$ represents the depth uncertainty estimated from the stereo matching process. The depth uncertainty is computed based on the reliability of stereo depth estimation, with higher uncertainty assigned to regions where depth estimation is less reliable, derived from both local disparity gradient consistency and image gradient information to provide robust depth estimates across different scene regions. Specifically, the depth uncertainty is formulated as,

(13) $$\sigma_{depth}=\frac{d_{e}\left(x\right)}{\tau_{e}}\left(1-\frac{{∥\nabla D(x)∥}_{2}}{P_{90}\;\left({∥\nabla D∥}_{2}\right)}\right)$$

where x=(x,y) denotes the 2D pixel coordinate in the image plane, ${∥\cdot ∥}_{2}$ denotes the Euclidean norm of the disparity gradient measuring local disparity variation, $P_{90}\;\left(\cdot \right)$ denotes the 90th percentile of disparity gradient magnitudes over the entire image used as a normalization factor, $d_{e}\left(x\right)$ denotes the Euclidean distance from pixel x to the nearest image edge extracted using the Canny operator, and $\tau_{e}$ denotes a normalization constant controlling the influence range of image edges on the confidence value.

The semantic consistency loss $L_{sem}^{t}$ is formulated as a multi-class cross-entropy (softmax + CE),

(14) $$L_{sem}^{t}=-\sum_{c=1}^{N_{sem}}S_{c}\log\left({\hat{S}}_{c}\right)$$

where *S* is a one-hot semantic label and $\hat{S}$ is the decoded class-probability vector at each pixel.

The composite mapping loss $L_{mapping}^{t}$ incorporates awareness of unobservable regions while jointly optimizing color, geometric, and semantic consistency,

(15) $$L_{mapping}^{t}=M_{unobs}\left(\lambda_{c}^{m}L_{color}^{t}+\lambda_{g}^{m}L_{geo}^{t}+\lambda_{s}^{m}L_{sem}^{t}\right)$$

where $\lambda_{c}^{m}=1.0$, $\lambda_{g}^{m}=0.5$, and $\lambda_{s}^{m}=0.2$ are the color, geometric, and semantic consistency loss weights, respectively.

### 3.4. Tracking and Keyframe Selection

Given a Gaussian scene representation $G_{t}$, the camera pose initialization follows a constant-velocity model in SE(3) [24],

(16) $$T_{t+1}^{\left(0\right)}=T_{t}\exp\;\left(\log\;\left(T_{t-1}^{-1}T_{t}\right)\right).$$

In implementation, this is approximated as first-order extrapolation of rotation and translation parameters:

(17) $$q_{t+1}^{\left(0\right)}=\mathrm{norm}\;\left(q_{t}+\left(q_{t}-q_{t-1}\right)\right),\;t_{t+1}^{\left(0\right)}=t_{t}+\left(t_{t}-t_{t-1}\right),$$

where *q* is the camera quaternion and t is translation. To ensure accurate camera pose estimation, only those rendered pixels with reliable depth information are factored into the tracking loss function. Then the camera pose is updated iteratively by gradient descent optimization through differentiably rendering color, depth, and semantic maps, minimizing the following loss function,

(18) $$L_{tracking}=\left(\hat{O}>\tau_{unobs}\right)\left(\lambda_{c}^{t}L_{color}^{t}+\lambda_{g}^{t}L_{geo}^{t}+\lambda_{s}^{t}L_{sem}^{t}\right)$$

where $L_{color}^{t}=L_{1}\left(\hat{C},C\right)$ simply employs the $l_{1}$-norm between the rendered image and the ground truth, $\lambda_{c}^{t}=1.0$, $\lambda_{d}^{t}=0.5$, and $\lambda_{s}^{t}=0.01$ are the weights for each term.

To address the limitations of fixed-interval keyframe selection, we propose a hybrid keyframe selection strategy that combines adaptive motion-based selection with a fixed-interval fallback mechanism. The motion-aware selection computes the relative transformation between the current frame and the most recently selected keyframe, estimating rotation angle θ and translation distance *d*,

(19) $$\theta =\arccos\left(\frac{\mathrm{tr}(R_{rel})-1}{2}\right),\;d={∥t_{rel}∥}_{2}$$

where $R_{rel}$ and $t_{rel}$ are the rotation matrix and translation vector of the relative transformation, respectively. If either $\theta >\theta_{\mathrm{th}}=10^{\circ }$ or $d>d_{\mathrm{th}}=$ 0.1 m, the current frame is selected as a keyframe. Otherwise, the method defaults to fixed-interval selection for temporal regularity.

Additionally, special handling ensures robust initialization and completion: the first and final frames are always selected as keyframes. We implement random keyframe sampling during optimization, selecting a subset of keyframes each iteration to reduce computational load while maintaining global map consistency.

## 4. Experiment

### 4.1. Experimental Setup

Datasets. To compare with other neural explicit SLAM methods, we evaluate our method on both the synthetic dataset (TartanAir [25]) and the real-world dataset (EuRoC [26]). Both datasets provide stereo image sequences along with ground truth camera poses. Due to memory constraints, each sequence runs a fixed number of frames to ensure a comprehensive evaluation.

Evaluation Metrics. Following [5,18,19], we use ATE for pose evaluation, PSNR/ SSIM [27]/LPIPS [28] for rendering quality, and mIoU for semantic accuracy (from dataset-provided annotations used only for evaluation). We also report Depth L1 between rendered depth and stereo-estimated depth as an internal geometric-consistency indicator, not as an independent depth benchmark.

Baseline. For tracking and mapping, we compare with classical stereo visual odometry such as SDSO [29], VINS-Fusion [30] and ORB-SLAM3 [31], as well as several recent representative 3DGS-based methods, including MonoGS [16], Photo-SLAM [32], SplaTAM [5] and GS3LAM [18]. For semantic reconstruction, our method is compared with NICE-SLAM [12], GS3LAM [18], and SGS-SLAM [33]. We explicitly distinguish baseline result sources as: R (re-run by us on the RTX 4090 platform under matched input settings) and P (numbers reported in the original papers when full reproducibility assets are unavailable). R: {SGS-SLAM, SplaTAM, GS3LAM, MonoGS, NICE-SLAM, SDSO, ORB-SLAM3, VINS-Fusion}; P: {Photo-SLAM not re-run due to reproducibility-asset limitations}.

Implementation Details. Experiments are run on an NVIDIA RTX 4090 GPU (24 GB) and a 16-core Intel Xeon(R) Gold 6430 CPU. The stereo network is pre-trained on SceneFlow [34] and fine-tuned only on designated training splits (evaluation sequences excluded), then kept frozen during SLAM inference; the semantic network is pre-trained on SA-1B [23]. Unless stated otherwise, StereoGS-SLAM results are averaged over 10 random seeds and reported as mean ± standard deviation, with 95% CI ($\mu \pm 1.96\sigma /\sqrt{10}$) provided in supplementary statistics. Runtime is profiled as ms/frame and FPS at 640 × 480 (TartanAir) and 752 × 480 (EuRoC). Tracking uses learning rates $\left\{4\times 10^{-4},2\times 10^{-3}\right\}$ for camera rotation/translation and 0 for Gaussian parameters; mapping uses 0.0001 (means3D), 0.0025 (RGB/semantic), 0.001 (rotation/log-scales), and 0.05 (opacity), with semantic-decoder Adam LR $5\times 10^{-4}$. Pruning is enabled every 20 iterations with an opacity threshold of 0.005. Full execution order, trigger conditions, pseudocode, and default parameters are provided in Appendix A (Algorithm A1, Table A1).

### 4.2. Rendering Evaluation

**Figure 2:**
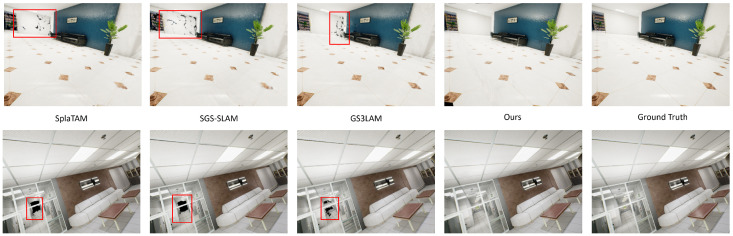
Qualitative rendering comparison on TartanAir “Hospital” and “Office” scenes. Our StereoGS-SLAM achieves improved visual quality with sharper object boundaries and better geometric fidelity compared with baseline methods, particularly in regions with large depth variations.

Table 1 compares rendering performance on TartanAir. Although competing methods leverage ground-truth depth, our stereo method achieves the best overall performance across sequences. These gains are attributed to adaptive scale refinement and semantic-aware optimization. Figure 2 provides a qualitative comparison, where StereoGS-SLAM yields sharper boundaries and fewer artifacts under large depth variations.

Table 2 reports the quantitative evaluation of reconstruction quality on the EuRoC dataset. The proposed method shows consistent improvements across most sequences, demonstrating strong robustness and generalization in real-world scenarios. Since Photo-SLAM provides results for only four sequences, our evaluation is restricted to those subsets. These results indicate that our system handles challenging real-world conditions, including dynamic environments and textureless surfaces frequently encountered in practical robotic applications.

Stereo vs. RGB-D Depth Quality. When comparing against RGB-D baselines, it is important to account for the sensing modality. RGB-D sensors provide direct depth measurements, but their depth accuracy and resolution typically degrade with increasing range [35]. Stereo depth is inferred from disparity; small disparity errors can induce depth errors that grow with distance, and matching is particularly difficult in textureless, occluded, or illumination-varying regions [36,37]. Consequently, RGB-D pipelines may benefit from higher-quality depth inputs under favorable conditions, while passive stereo methods can be disadvantaged in challenging scenes. Our results indicate that StereoGS-SLAM remains competitive despite operating under this more demanding stereo-depth setting.

### 4.3. Tracking Evaluation

Table 3 summarizes the tracking performance on the TartanAir dataset. We compare the proposed StereoGS-SLAM with widely used traditional SLAM methods and recent 3DGS-based SLAM systems. For re-running traditional SLAM baselines, loop closure detection is disabled to reduce pipeline-side advantages under different back-end settings. Both SDSO and VINS-Fusion experience tracking failures due to their high sensitivity to photometric variations within the scenes. Benefiting from the incorporation of spatial stereo constraints during the tracking stage, StereoGS-SLAM achieves higher tracking accuracy compared with other 3DGS-based methods on our tested sequences.

**Table 1:** Qualitative Rendering Evaluation on TartanAir (mean ± std).

Method	Metric	Hospital			Car Welding			Office			Avg.
		P000	P001	P002	P001	P002	P004	P000	P001	P002	
SplaTAM [5]	PSNR ↑	26.255 ± 0.144	20.69 ± 0.091	26.161 ± 0.226	14.438 ± 0.005	14.983 ± 0.193	14.048 ± 0.221	23.754 ± 0.109	26.138 ± 0.272	20.421 ± 0.278	20.765 ± 0.171
	SSIM ↑	0.907 ± 0.008	0.861 ± 0.009	0.956 ± 0.008	0.581 ± 0.004	0.77 ± 0.011	0.505 ± 0.010	0.884 ± 0.010	0.944 ± 0.011	0.824 ± 0.006	0.804 ± 0.009
	LPIPS ↓	0.147 ± 0.011	0.277 ± 0.016	0.072 ± 0.017	0.482 ± 0.012	0.241 ± 0.007	0.478 ± 0.015	0.215 ± 0.014	0.115 ± 0.019	0.226 ± 0.0013	0.250 ± 0.013
MonoGS [16]	PSNR ↑	22.621 ± 0.281	20.627 ± 0.128	28.375 ± 0.289	16.002 ± 0.255	19.911 ± 0.191	17.22 ± 0.204	25.596 ± 0.006	25.67 ± 0.177	20.018 ± 0.088	21.782 ± 0.180
	SSIM ↑	0.835 ± 0.012	0.688 ± 0.018	0.841 ± 0.012	0.488 ± 0.011	0.641 ± 0.008	0.411 ± 0.017	0.876 ± 0.019	0.869 ± 0.007	0.712 ± 0.005	0.707 ± 0.012
	LPIPS ↓	0.363 ± 0.013	0.497 ± 0.015	0.162 ± 0.011	0.53 ± 0.012	0.423 ± 0.022	0.37 ± 0.014	0.244 ± 0.015	0.222 ± 0.013	0.382 ± 0.015	0.355 ± 0.014
SGS-SLAM [33]	PSNR ↑	26.714 ± 0.044	20.998 ± 0.189	26.124 ± 0.012	13.348 ± 0.157	15.097 ± 0.267	14.168 ± 0.238	23.248 ± 0.261	26.158 ± 0.165	19.982 ± 0.188	20.649 ± 0.169
	SSIM ↑	0.917 ± 0.007	0.880 ± 0.019	0.958 ± 0.012	0.565 ± 0.008	0.771 ± 0.011	0.510 ± 0.015	0.862 ± 0.018	0.940 ± 0.016	0.805 ± 0.015	0.801 ± 0.013
	LPIPS ↓	0.138 ± 0.016	0.255 ± 0.006	0.070 ± 0.004	0.492 ± 0.004	0.241 ± 0.011	0.474 ± 0.019	0.245 ± 0.011	0.120 ± 0.016	0.249 ± 0.016	0.254 ± 0.011
GS3LAM [18]	PSNR ↑	27.988 ± 0.139	20.025 ± 0.162	27.741 ± 0.244	14.935 ± 0.210	16.927 ± 0.223	18.072 ± 0.056	25.084 ± 0.131	26.817 ± 0.253	22.17 ± 0.160	22.195 ± 0.175
	SSIM ↑	0.939 ± 0.013	0.849 ± 0.007	0.953 ± 0.008	0.565 ± 0.005	0.837 ± 0.018	0.743 ± 0.013	0.915 ± 0.007	0.948 ± 0.004	0.859 ± 0.004	0.845 ± 0.009
	LPIPS ↓	0.116 ± 0.006	0.28 ± 0.003	0.067 ± 0.004	0.494 ± 0.017	0.188 ± 0.017	0.276 ± 0.005	0.171 ± 0.003	0.108 ± 0.012	0.185 ± 0.015	0.209 ± 0.009
Ours	PSNR ↑	29.630 ± 0.281	24.084 ± 0.166	28.867 ± 0.197	19.268 ± 0.042	22.102 ± 0.183	21.080 ± 0.051	26.032 ± 0.060	28.744 ± 0.104	22.125 ± 0.114	24.659 ± 0.102
	SSIM↑	0.962 ± 0.003	0.945 ± 0.004	0.956 ± 0.015	0.731 ± 0.005	0.852 ± 0.011	0.866 ± 0.012	0.932 ± 0.004	0.955 ± 0.007	0.821 ± 0.004	0.891 ± 0.007
	LPIPS ↓	0.090 ± 0.008	0.212 ± 0.004	0.101 ± 0.029	0.389 ± 0.015	0.222 ± 0.012	0.211 ± 0.006	0.151 ± 0.020	0.081 ± 0.015	0.221 ± 0.021	0.186 ± 0.014

Note: Green highlighting indicates best performance. Yellow highlighting indicates second-best performance. ↑ denotes higher-is-better and ↓ denotes lower-is-better. SplaTAM, SGS-SLAM, GS3LAM run in RGB-D mode; MonoGS runs in stereo mode. The evaluation is conducted on three scenes: Hospital, Car Welding, and Office, with three different trajectory sequences (e.g., P000, P001, P002) selected from each scene.

**Table 2:** Qualitative Rendering Evaluation on EuRoC (mean ± std).

Method	Metric	MH01	MH02	V101	V201	Avg.
SplaTAM [5]	PSNR ↑	13.395 ± 0.252	15.322 ± 0.015	19.578 ± 0.195	20.045 ± 0.276	17.085 ± 0.185
	SSIM ↑	0.575 ± 0.018	0.635 ± 0.011	0.800 ± 0.012	0.858 ± 0.009	0.717 ± 0.013
	LPIPS ↓	0.347 ± 0.026	0.345 ± 0.015	0.161 ± 0.016	0.126 ± 0.028	0.245 ± 0.021
Photo-SLAM [32]	PSNR ↑	13.952	14.201	17.069	15.677	15.225
	SSIM ↑	0.420	0.430	0.618	0.622	0.523
	LPIPS ↓	0.366	0.356	0.266	0.323	0.328
MonoGS [16]	PSNR ↑	17.783 ± 0.020	15.721 ± 0.003	16.332 ± 0.288	22.384 ± 0.244	18.055 ± 0.139
	SSIM ↑	0.709 ± 0.008	0.709 ± 0.020	0.767 ± 0.011	0.893 ± 0.003	0.770 ± 0.011
	LPIPS ↓	0.281 ± 0.024	0.227 ± 0.010	0.281 ± 0.025	0.210 ± 0.014	0.250 ± 0.018
GS3LAM [18]	PSNR ↑	16.870 ± 0.113	18.002 ± 0.052	19.186 ± 0.093	20.749 ± 0.118	18.702 ± 0.094
	SSIM ↑	0.679 ± 0.004	0.776 ± 0.010	0.798 ± 0.015	0.853 ± 0.018	0.777 ± 0.012
	LPIPS ↓	0.287 ± 0.016	0.265 ± 0.014	0.171 ± 0.004	0.125 ± 0.015	0.212 ± 0.012
Ours	PSNR ↑	18.722 ± 0.159	19.154 ± 0.270	20.298 ± 0.258	22.505 ± 0.183	20.170 ± 0.217
	SSIM ↑	0.841 ± 0.012	0.759 ± 0.015	0.810 ± 0.010	0.907 ± 0.008	0.829 ± 0.011
	LPIPS ↓	0.214 ± 0.009	0.257 ± 0.012	0.134 ± 0.007	0.126 ± 0.006	0.183 ± 0.009

Note: Green highlighting indicates best performance. Yellow highlighting indicates second-best performance. ↑ denotes higher-is-better and ↓ denotes lower-is-better. SplaTAM, Photo-SLAM, and GS3LAM run in RGB-D mode, while MonoGS runs in stereo mode. Some baselines are re-run on our hardware while others are paper-reported due to reproducibility limitations.

### 4.4. Semantic Evaluation

Table 4 presents the quantitative semantic evaluation results in comparison with existing neural semantic SLAM approaches. StereoGS-SLAM achieves strong performance and improves over prior baselines by more than 2% on average. As shown in the table, 3DGS-based methods generally outperform NeRF-based approaches in semantic reconstruction. This is primarily because the explicit Gaussian representation can accurately delineate object boundaries, producing more precise and spatially consistent 3D semantic segmentation. Compared with other 3DGS-based approaches, StereoGS-SLAM further improves semantic reconstruction quality. This improvement arises from the proposed adaptive scene depth estimation mechanism, which dynamically adjusts Gaussian scales to better capture scene geometry. By reducing uncertainty at object surfaces and boundaries, the system achieves stronger geometric-semantic alignment. Additionally, the introduced random sampling-based keyframe mapping strategy reduces optimization bias and improves global semantic consistency.

### 4.5. Ablation Study

**Figure 3:**
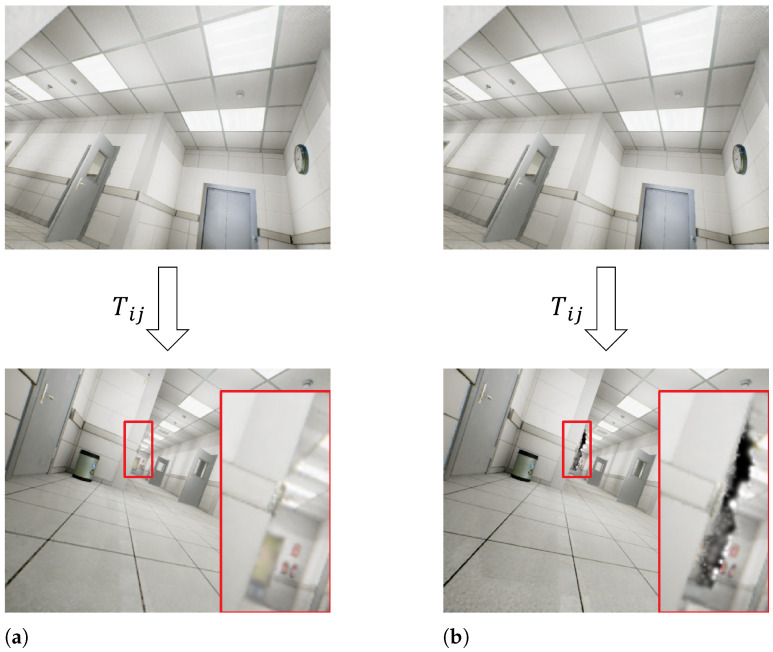
The ablation study on adaptive scene depth estimation on TartanAir “Hospital”. When the depth of the scene changes with camera viewpoint rotation, adaptive scene depth estimation can help to refine Gaussian scales. (**a**) w/ adaptive scene depth estimation. (**b**) w/o adaptive scene depth estimation.

Adaptive Scene Depth Estimation. We isolate the effect of the adaptive scene depth estimation (ASDE) by disabling only this module while keeping all other components unchanged and compare the results with the full system. As illustrated in Figure 3, removing adaptive depth estimation leads to enlarged Gaussian scales near object boundaries, resulting in blurred contours and reduced geometric precision. Quantitative results in Table 5 further confirm this degradation: PSNR decreases by 2.199 dB, SSIM drops by 0.04, and LPIPS increases by 0.03. This indicates that the system becomes less capable of preserving high-frequency structural detail. Moreover, removing dynamic scale adaptation results in a 0.98 cm increase in ATE RMSE, highlighting its importance for stable camera pose estimation across different depth ranges.

**Figure 4:**
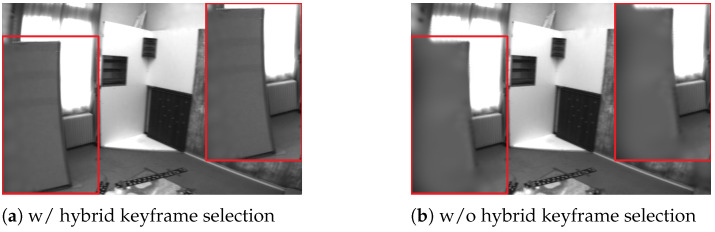
The ablation study on hybrid keyframe selection on EuRoC “V102”. Without the hybrid keyframe selection strategy, the system exhibits poorer scene coherence and reduced rendering quality.

Hybrid Keyframe Selection Ablation. We ablate the hybrid keyframe strategy to assess its impact on map consistency and tracking. As shown in Figure 4, removing the strategy yields uneven keyframe coverage and biased sampling, causing over-optimized local regions and under-updated areas. Table 5 shows clear degradations and a higher ATE RMSE, indicating reduced scene coherence and increased pose drift. These results confirm that the hybrid strategy mitigates optimization bias and improves long-term stability.

## 5. Conclusions

We propose StereoGS-SLAM, a stereo semantic SLAM system that leverages 3DGS for explicit scene representation. Unlike existing approaches, the proposed method operates solely on passive RGB stereo pairs without relying on active depth sensors, thereby improving its applicability in real-world robotic environments. The core technical contributions include an adaptive scene depth estimation strategy that dynamically adjusts Gaussian scales, and a hybrid keyframe selection mechanism that improves keyframe diversity while maintaining computational efficiency. Comprehensive evaluations on the TartanAir and EuRoC datasets show that StereoGS-SLAM achieves competitive performance in camera pose estimation, rendering fidelity, and semantic reconstruction compared with recent 3DGS-based SLAM systems. Our contributions extend the 3DGS-SLAM pipeline by incorporating stereo depth-driven scale adaptation and more stable keyframe optimization, thereby improving both geometric precision and semantic consistency.

## 6. Future Work

Future work can proceed along several directions. First, improving stereo depth reliability in low-texture or reflective regions could further stabilize mapping, for example, by incorporating uncertainty-aware weighting or temporal consistency in depth estimation. Second, long-term semantic consistency can be strengthened with lightweight temporal regularization and more effective keyframe management. Third, extending the system to additional sensing modalities may improve robustness under fast motion and challenging lighting. Finally, the overall cost still increases as the map expands over long trajectories due to the growth of $N_{g}$; more aggressive map compression and adaptive Gaussian pruning are promising for scalability while preserving rendering and semantic quality.   

## Figures and Tables

**Table 3:** Tracking performance on TartanAir (ATE RMSE [cm]).

Method	Hospital	Car Welding	Office	Avg.
SDSO [29]	12.102 ± 1.370	x	x	-
ORB-SLAM3 [31]	4.273 ± 0.529	16.940 ± 0.831	6.330 ± 0.742	9.071 ± 0.701
VINS-Fusion [30]	6.910 ± 0.806	x	9.680 ± 0.813	-
SplaTAM [5]	4.010 ± 0.444	12.195 ± 0.984	3.460 ± 0.512	6.555 ± 0.647
SGS-SLAM [33]	2.017 ± 0.232	12.010 ± 0.345	4.257 ± 0.298	6.095 ± 0.292
MonoGS [16]	1.153 ± 0.137	15.077 ± 0.421	6.366 ± 0.389	7.532 ± 0.316
GS3LAM [18]	2.390 ± 0.276	13.413 ± 0.354	8.860 ± 0.412	8.221 ± 0.347
Ours	0.510 ± 0.012	10.930 ± 0.015	4.190 ± 0.018	5.210 ± 0.045

Note: Green highlighting indicates best performance. Yellow highlighting indicates second-best performance. “x” indicates the method failed to track the entire sequence. Both ORB-SLAM3 and VINS-Fusion are reported in Stereo mode without loop closure.

**Table 4:** Quantitative comparison of semantic reconstruction accuracy on TartanAir (mIoU [%]).

Method	Hospital	Car Welding	Office	Avg.
NICE-SLAM [12]	65.32 ± 1.380	61.07 ± 0.807	60.27 ± 1.298	62.22 ± 1.162
SGS-SLAM [33]	68.47 ± 0.809	69.80 ± 1.360	66.50 ± 0.693	68.26 ± 0.954
GS3LAM [18]	70.50 ± 1.588	67.14 ± 0.925	68.92 ± 0.829	68.85 ± 1.114
Ours	75.21 ± 0.451	74.79 ± 0.382	69.57 ± 0.427	73.19 ± 0.420

Note: Green highlighting indicates best performance. Yellow highlighting indicates second-best performance.

**Table 5:** The ablation study on TartanAir Hospital.

Method	Metrics				
	PSNR	SSIM	LPIPS	ATE	mIoU
w/o ASDE	26.545 ± 0.123	0.915 ± 0.010	0.111 ± 0.008	5.170 ± 0.200	58.920 ± 0.450
w/o HKFS	27.010 ± 0.130	0.940 ± 0.012	0.105 ± 0.006	6.452 ± 0.210	68.030 ± 0.480
Ours	28.744 ± 0.140	0.955 ± 0.005	0.081 ± 0.004	4.190 ± 0.180	69.570 ± 0.420

Note: Green highlighting indicates best performance.

## Data Availability

The datasets used in this study (TartanAir and EuRoC) are publicly available. Although the full implementation is currently not publicly released, this manuscript provides a reproducibility-oriented specification, including expanded pseudocode, explicit optimization schedules, complete key hyperparameters, and dataset protocol settings intended to support independent replication.

## Appendix A. Reproducibility Specification

### Appendix A.1. Default Reproducibility Parameters

Table A1 summarizes the default optimization, scheduling, and pruning hyperparameters used in the main experiments for direct protocol reproduction.

**Table A1:** Default Reproducibility Parameters Used in Main Experiments.

Category	Parameter	Value
Global	Number of runs	10 seeds
Global	Seed (default)	1
Global	map_every/keyframe_every	1/5
Global	eval_every/checkpoint_interval	5/100
Tracking	Iterations per frame	360
Tracking	Pose learning rates	rot: $4\times 10^{-4}$, trans: $2\times 10^{-3}$
Tracking	Gaussian parameter learning rates	0 (fixed during tracking)
Tracking	Alpha mask threshold	0.99
Mapping	Iterations per frame	150
Mapping	First-frame mapping iterations	1000 (TartanAir), 1200 (EuRoC)
Mapping	Semantic decoder LR	$5\times 10^{-4}$
Mapping	Gaussian learning rates	m3D: $1\times 10^{-4}$; RGB/sem: $2.5\times 10^{-3}$; rot/scale: $1\times 10^{-3}$; opac: $5\times 10^{-2}$
Mapping	Random keyframe sampling interval	5
Mapping	New-Gaussian threshold (densify_thres)	0.1
Pruning	prune_every/stop_after	20/20
Pruning	Opacity threshold	0.005

### Appendix A.2. Runtime and GPU Memory Analysis

Table A2 summarizes runtime and GPU memory usage on the RTX 4090 platform with a direct comparison to GS3LAM. Runtime is reported as ms/Iteration and s/frame, while memory is reported in GB. In the current version, we do not include a dedicated memory-compression module; therefore, GPU memory is expected to increase with the number of Gaussians over long trajectories.

**Table A2:** Runtime and GPU memory statistics on RTX 4090.

Category	Metric	Ours	GS3LAM	Unit
Runtime	Tracking iteration time	59.17	47.69	ms/Iteration
	Mapping iteration time	38.36	26.09	ms/Iteration
	Tracking frame time	3.18	2.15	s/frame
	Mapping frame time	4.72	3.84	s/frame
	FPS (tracking/mapping)	0.314/0.212	0.465/0.260	FPS
GPU Memory	Average allocated	21.13	22.80	GB

### Appendix A.3. Algorithmic Specification

To make the implementation behavior fully traceable, we provide Algorithm A1 as an execution-level specification of the full pipeline. The procedure explicitly defines initialization, per-frame tracking updates, mapping trigger conditions, keyframe insertion policy, Gaussian add/prune operations, checkpoint scheduling, and final evaluation/export steps. It also makes stochastic components reproducible by fixing random seeds and documenting random keyframe sampling conditions. Together with the parameter table in this appendix, this algorithm is intended to support independent re-implementation under matched protocol settings.

**Algorithm A1** StereoGS-SLAM Detailed Execution Procedure
Require: Stereo stream ${\left\{I_{t}^{L},I_{t}^{R}\right\}}_{t=0}^{T-1}$, intrinsics K, seed *s*, config Θ Ensure: Estimated poses $\left\{T_{t}\right\}$, Gaussian map G 1: Set random seeds using *s*; initialize first-frame Gaussians and map states 2: Initialize empty keyframe set K←∅ 3: **for** t=0 to T−1 **do** 4: Load $\left(I_{t}^{L},I_{t}^{R}\right)$ and semantic supervision $S_{t}$ 5: Estimate disparity and depth $D_{t}$; construct current-frame data tuple 6: **if** t>0 **then** 7: Initialize $T_{t}$ by forward propagation from previous poses 8: **for** i=1 to $N_{\mathrm{track}}$ **do** 9: Render $\left(\hat{C},\hat{D},\hat{S},\hat{O}\right)$ from $\left(G,T_{t}\right)$ 10: Compute tracking loss and update camera parameters only 11: Keep best pose candidate by minimum tracking loss 12: **end for** 13: **end if** 14: **if** t=0 or $\left(t+1\right)\;\mathrm{mod}\;m_{\mathrm{map}}=0$ **then** 15: **if** t>0 and add-new-Gaussians is enabled **then** 16: Build non-presence mask using alpha/depth inconsistency criteria 17: Add Gaussians from masked pixels via depth unprojection 18: Update adaptive scene radius and smooth by exponential moving average 19: **end if** 20: Set mapping iterations $N_{\mathrm{map}}^{*}$ (first frame uses larger value) 21: **for** j=1 to $N_{\mathrm{map}}^{*}$ **do** 22: **if** t=0 or $j\;\mathrm{mod}\;r_{\mathrm{rskm}}=0$ **then** 23: Use current frame for mapping 24: **else** 25: Randomly sample one keyframe from K for mapping 26: **end if** 27: Compute mapping loss (color + geometry + semantics + regularization) 28: Update Gaussian and semantic decoder parameters 29: Apply Gaussian pruning/densification if enabled 30: **end for** 31: **end if** 32: **if** t=0 or $\left(t+1\right)\;\mathrm{mod}\;m_{\mathrm{kf}}=0$ or t=T−2 **then** 33: Insert frame *t* into keyframe set K 34: **end if** 35: **if** checkpointing enabled and $t\;\mathrm{mod}\;m_{\mathrm{ckpt}}=0$ **then** 36: Save intermediate parameters 37: **end if** 38: **end for** 39: Run final evaluation and export trajectories/map parameters
